# Foundational digital literacy training for frontline immunization officers: lessons from implementing the electronic stock management tool across selected comprehensive health centers in Sierra Leone

**DOI:** 10.3389/fdgth.2025.1673085

**Published:** 2025-12-19

**Authors:** Iniobong Ekong, Tom Sesay, Regina Samuels, Edward Foday, Francis Smart, Desmond Kangbai, Agazi Ameha, Vandana Joshi, Alhassan Mayei, Tessa Lennemann

**Affiliations:** 1 Digital Health Consultant, UNICEF, Freetown, Sierra Leone; 2 Ministry of Health, Directorate of Policy, Planning and Information, Government of Sierra Leone, Freetown, Sierra Leone; 3 Programme Manager, Expanded Programme on Immunization, Ministry of Health, Government of Sierra Leone, Freetown, Sierra Leone; 4 Health, Nutrition and HIV section, United Nations Children's Fund, Freetown, Sierra Leone; 5 GIZ Deutsche Gesellschaft für Internationale Zusammenarbeit, Freetown, Sierra Leone

**Keywords:** digital literacy, training needs analysis, competency framework, digitalization, e-readiness, digital transformation

## Abstract

**Background:**

Sierra Leone has advanced its digital health agenda. However, digital literacy among frontline health workers remains low, with over 82% reporting limited confidence in using digital tools. The health workforce also recorded the lowest digital health maturity score among all enablers in the WHO Global Digital Health Monitor, underscoring the need for workforce upskilling as a foundation for digital transformation.

**Objective:**

This paper describes the design, implementation, and outcomes of a foundational digital literacy training program for frontline health workers under the Digital Innovation in Pandemic Control (DIPC) project, aimed at improving readiness for digital tool adoption.

**Methods:**

A training needs analysis (TNA) aligned skill gaps with the competencies required for using the electronic Stock Management Tool (eSMT). Training modules were adapted from the European Commission's DigComp framework, contextualized for Sierra Leone, and delivered through a blended learning model. Post-training competency gains were assessed to determine effectiveness.

**Results (Implementation):**

Among 150 trained health workers, “high understanding” in basic computer literacy increased from 7.1% to 72.2%, while “low understanding” dropped from 65.9% to 9.2%. For computer troubleshooting skills, “high understanding” rose from 4.4% to 73.8%. Both courses showed large effect sizes (Cohen's *d* = 1.3–2.1), indicating substantial learning gains.

**Conclusions:**

Systematic digital literacy training, grounded in competency frameworks and contextual design, can substantially improve digital readiness among frontline health workers. Such interventions are essential foundations for sustainable digital transformation in health systems.

## Introduction

### Overview

Adoption of digital technologies in the health sector has witnessed rapid increase in recent years (1). The global COVID-19 pandemic threat has significantly contributed to this trend (2, 59). Though not without challenges, many LMICs continue to adopt these technologies to advance healthcare (3, 4). Digital technologies have no doubt shown promise in effectively addressing health systems challenges (5) informing their recommended scale-up to achieve greater impact (6, 7).

**Table 1:** Digital literacy scale.

Level	Points	Description
L1	0–5	No understanding
L2	6–10	Very low understanding
L3	11–15	Low understanding
L4	16–20	Average understanding
L5	21–25	High understanding
L6	26–30	Very high understanding

However, implementation outcomes in digital health initiatives are influenced not only by infrastructure, but also by factors such as institutional readiness, user confidence, and behavioral responses to new technologies (8). This underscores the need to consider human and organizational factors when assessing digital transformation. Successful adoption of digital health solutions depends on the systematic understanding of these dimensions and the integration of relevant theoretical frameworks throughout the design and implementation phases.

### Theoretical framework: technology acceptance, self-efficacy and e-readiness

Digital health adoption is shaped by complex interactions between individual, organizational, and contextual determinants. The Technology Acceptance Model (TAM) (9) posits that users' adoption decisions are influenced by perceived usefulness and perceived ease of use, which in turn affect behavioral intention and system use. In healthcare, these perceptions are strongly mediated by self-efficacy, the belief in one's ability to successfully perform technology-related tasks (10). Low self-efficacy has been linked to low confidence and resistance to digital innovation, while training interventions that improve competence and confidence strengthen both perceived ease of use and perceived usefulness (11).

Furthermore, the degree of e-readiness—the preparedness of individuals, institutions, and systems to adopt and sustain digital technologies (12), moderates these relationships. E-readiness encompasses access to digital infrastructure, leadership commitment, policies, and skills alignment. Integrating these theories provides a more holistic understanding of how workforce training can translate into actual technology use and institutional transformation, particularly in low-resource settings.

### Digital transformation

Efforts to unlock digital transformation gains are often hampered by digital literacy gaps among health workforce as evident during the COVID-19 pandemic (2, 11). Addressing these gaps through computer skills training alone are incapable of advancing digital transformation (13). For digital transformation to occur, a wide range of competencies must be applied to create new knowledge, innovate and communicate (14, 15). Healthcare professionals need digital readiness by acquiring these competencies (16). This differentiates digital literacy from computer literacy. Digital literacy involves the mastery of simple and practical skills required to be digitally intelligent and creative (60), and to effectively compete in the 21st century (17). This is critical in protecting against the associated risk of digital investments (18).

### Training needs analysis

Training Needs Analysis (TNA) is an essential first step in designing and developing effective training programmes (19). It aligns training goals with potential constraints faced by health workers and relevant competencies required to perform a specific task (20, 21). Many studies have demonstrated the positive linkages between TNA and achievement of training program goals (22, 23). This correlation stems from leveraging gaps identified from TNA, guided by the training goals for developing a contextual competency framework (24).

### Digital health competency framework development

Competency framework outlines the specific knowledge, skills and attitude required by job roles to successfully complete assigned tasks (25). Global digital literacy competency frameworks are well documented in literature. Each framework provides a reference targeting specific objectives – to achieve SDG Indicator 4.4.2 (26), foster emerging digital economies (27) and understand child-related digital literacy needs (60). The European Commission's Digital literacy framework (DigCom) provides 5 adaptable conceptual learning models for citizens and non-IT professionals (25). However, the Global Digital Health Literacy competency framework is currently under development (28).

### Novelty and research gap

While several studies have explored digital health literacy, they have largely focused on non-professional populations and academic or adolescent cohorts rather than on frontline health workers operating in real health system contexts. For example (29), assessed digital health literacy among students in Iran using a cross-sectional design but did not evaluate structured training interventions. Similarly (30),co-designed an educational resource for adolescents but did not assess its operational utility in healthcare delivery (31). Examined the relationship between digital literacy, trust, and health anxiety, yet their scope remained limited to non-clinical environments (10). Demonstrated that self-efficacy mediates the relationship between digital literacy and self-management behaviors, but their work was restricted to college students and lacked application to professional healthcare roles. Other related studies have similarly concentrated on general populations outside formal health system operations.

In contrast, this study is the first to document an applied digital literacy intervention targeted at frontline immunization officers in low-resource settings, integrating a competency-based training framework with operational digital tools deployed for pandemic control. Unlike previous works, it combines pre-deployment readiness assessment, context-specific skills training, and evaluation of post-training knowledge gains within a live national health project. This approach addresses the critical implementation gap between digital literacy theory and operational practice, contributing new empirical evidence on how structured training interventions can strengthen workforce preparedness and digital transformation in LMIC health systems.

### Blended learning

Appropriate training delivery approach is crucial to ensuring timeliness and addressing diverse learning needs (32, 33). Blended learning involves a combination of face-to-face direct facilitation, and self-paced online learning (34, 35). Previous studies have shown its ability to compensate the disadvantages of traditional learning and online learning (36), improve performance and critical thinking (37), promote skill development (38), enhance self-management in learning and motivation (39), and provide a satisfactory learning experience (40).

### Knowledge evaluation

Knowledge evaluation measures how well frontline health workers' skills match the tasks required to effectively operate a digital health application (41). This is crucial to determining the effectiveness of the training programme. However, post training knowledge gains were shown to depreciate significantly over time justifying the need for continuous training (42). Moreso, the availability of lifelong learning opportunities significantly contributes to the effectiveness of any digital literacy training program (43).

### Change management

Change management is a system of providing continuous end user support to address potential risks associated with adoption and use of new technologies (44). Adoption risks include unintended consequences arising from new digital culture and resulting in resistance (45). Many studies have linked unsatisfactory end user experience with resistance to change (46–49).

### Digital innovation in pandemic control (DIPC) project

The Sierra Leone Digital Innovation in Pandemic Control (DIPC) commenced in 2023 as part of post COVID-19 resilience building. It aimed to improve vaccine stock management and prevent stock-outs and wastages from expiry (50). Consequently, the electronic Stock Management Tool (eSMT) application was deployed across 96 CHCs in 4 selected Districts.

This project faced potential threats from observed digital literacy gaps among intended end users - frontline immunization officers. Prior to DIPC, workforce had the lowest digital health maturity score amongst all digital health enablers (61). This was attributed to gaps in both pre-service and in-service digital literacy training (51). Existing trainings were often *ad hoc*, lecture-styled, and one-off without continuous support. This approach has limited impact on learning and performance (62). With increasing digitalization, health workers needed new skills to adopt digital technologies and improve health program performance.

Considering the importance of ensuring workforce readiness prior to deployment of digital technology (43), a national digital literacy gap assessment was conducted in 2022. This assessment revealed 82% of frontline health workers rated themselves as having low confidence level to effectively operate a computer device and utilize ICT applications without technical assistance (51). The same report revealed that minor troubleshooting issues significantly contributed to over 12.4% of computer devices found to be non-functional. Addressing this situation required an effective approach to building the critical mass of digitally skilled health workforce required to facilitate the DIPC project and support the digital transformation drive. This informed the design and implementation of the digital literacy skills training programme.

This paper elaborates the processes and lessons learned from implementing digital literacy skills intervention program in Sierra Leone as part of enhancing vaccine logistics, real-time data management, and digital transformation in healthcare in the post COVID-19 era.

### Objectives

Motivated by the opportunity to share lessons learned in addressing challenges of low digital skilling of health workforce, this paper highlights the processes and lessons learned in implementing a competency-based foundational digital literacy skills intervention program to support achievement of the DIPC project goals and overarching digital health vision in Sierra Leone (62).

## Methods

### Overview

The goal of our training program was to empower 150 frontline immunization officers at the 96 Comprehensive Health Centres (CHC) selected for the DIPC project with the minimum competency required to effectively use the eSMT application. A TNA was conducted to define required competencies, develop training modules and design the training approach.

### Training needs analysis

TNA involved a hierarchical approach utilized to analyze the competencies required for the DIPC training goals (21, 52). Current competency gaps were identified using a self-reporting technology confidence tool and responses from 338 frontline health workers. This tool has been effectively applied in multiple studies to align digital literacy requirements with operational tasks (41, 53). Assessment gaps were adapted to training needs and aligned with the DigCom 2.2 framework to define the necessary skills and proficiency levels, which informed the development of training modules validated by a digital literacy work group.

### Training module development

A multi-stakeholder digital literacy work group was set up at the Ministry of Health (MoH) to develop contextualized training modules. The group membership included technical officers from the Ministry of Communications, Technology and Information (MoCTI), the Directorate of Science, Technology and Innovation (DSTI) and development partners. Existing digital literacy training modules from these organizations were consolidated and enhanced using locally relevant content, interactive videos with local language narration, and self-paced sub-modules in alignment with multimedia principles (54).

### Training cascade

Seventeen National and District levels immunization supervisors and ICT officers were trained on both digital literacy skills training facilitation and coaching skills. They were then supported to cascade the training to 150 immunization officers across 96 PHC facilities and 20 district level immunization officers and supervisors across the four DIPC districts. A face-to-face, direct hands-on training was conducted over a 4-day period. Each trainee was provided with a laptop device and internet connectivity. Instructional materials were competency focused and interactive with in-course test questions, direct simulations and individual skills practice sessions. The basic computer literacy and internet skills training course was delivered first to lay the foundation for the basic troubleshooting course which followed thereafter.

### Competency gain evaluation

A self-evaluating, identical pre and post-test questionnaire comprising 30 objective questions adapted from both training modules was administered to each of the 150 trainees. Only 139 and 142 trainees successfully completed both pre and post-test evaluation assessments for both courses respectively. Each correct answer was assigned 1 point while a wrong answer was assigned 0 point. The minimum and maximum possible scores for each participant ranged from 0 to 30 points respectively. A tool for measuring competency gain, based on the Digital Literacy Scale (DLS) shown in Table 1 and proven effective in assessing digital competency improvements, was adapted for use (11, 18, 55). To ensure cultural relevance and measurement accuracy, the adapted tool was reviewed and validated by the Ministry of Health and key national stakeholders before implementation. The adapted DLS was then used to grade the scores into six levels of understanding ranging from “no understanding” to “very high understanding”. The changes in the level of understanding between pre- and post-tests were compared to determine the competency gain for both courses. Post-course evaluation feedback survey was conducted at the end of both modules to measure training relevance and satisfaction.

### Change management

Continuous onsite coaching and mentoring support was provided to end users in addition to the blended learning approach adopted. We trained National and District levels coaches and supported them to conduct monthly on-the-job skills reinforcement coaching after the initial hands-on training. This aimed to facilitate the shift to a digital culture and shorten the learning curve for the eSMT application. Performance coaching technique was adopted due to its effectiveness in addressing both end-users learning barriers and meeting program performance goals (56). Additionally, lessons learned in previous coaching sessions were reflected upon and end users were actively involved in deciding the dates, duration and discussion for each coaching visit. This is crucial to achieving successful coaching (57). A virtual help desk platform was also created on WhatsApp to provide offsite remote support after the training and in between coaching visits.

## Results

### Training needs analysis

#### Confidence level assessment for identified tasks

**Figure 1:**
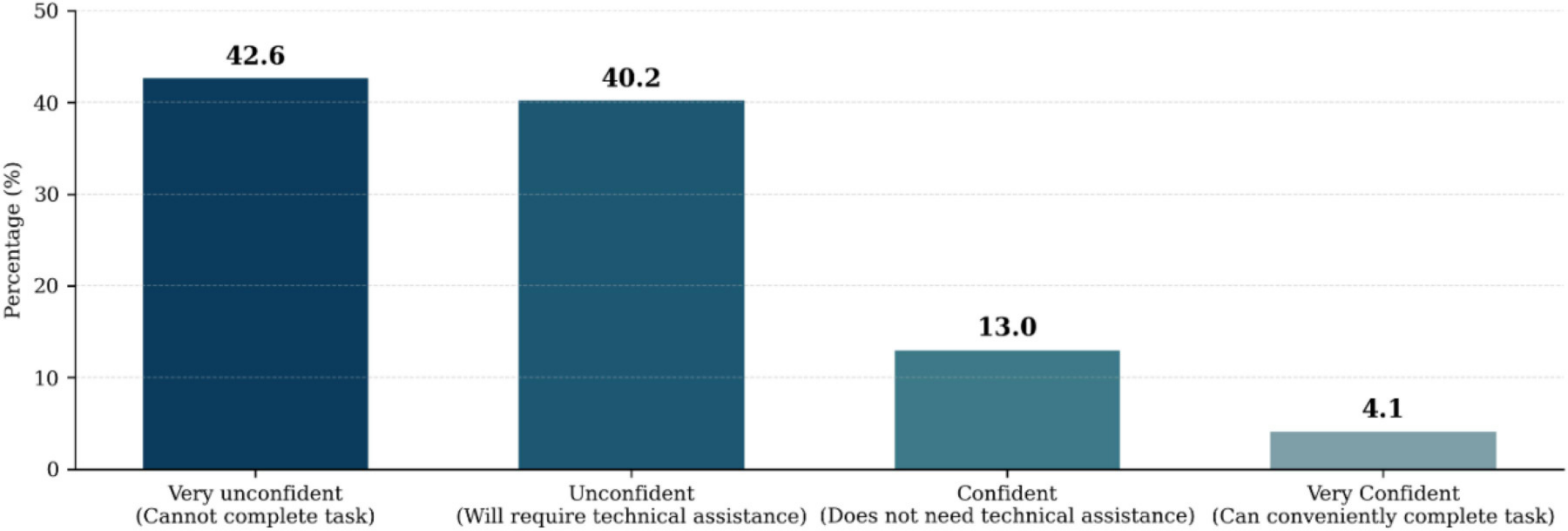
Self-reported pre-training digital competency confidence levels among healthcare workers.

A total of 338 healthcare professionals from 14 primary and secondary healthcare facilities completed the self-reported confidence level assessment tool regarding their ability to perform basic computer tasks. Participants indicated their comfort level with tasks such as basic device management, application navigation, document management, internet browsing, and email communication. The respondent demographics included Nurses (51.2%), Community Health Officers/Assistants (26.9%), Laboratory Scientists/Technicians (6.5%), Administrative Staff (4.1%), Doctors (11%), Pharmacists (2.1%), and Data Clerks/Monitoring and Evaluation Officers (5.9%). Analysis of the results revealed that a significant majority, 82.8% of healthcare workers reported feeling unconfident in performing basic computer tasks (Figure 1). Meanwhile, 13% expressed confidence, and only 4.2% felt very confident in their abilities. These findings highlighted a substantial need for targeted training programs to enhance digital competency among healthcare workers.

### Training module development

The training modules were developed by aligning training requirements identified from the TNA with the DigCom 2.2 digital competency framework (Table 2). This informed the proficiency level and content for the seventeen training modules developed for both Basic Computer Literacy and Internet Skills (BCLIS) and Basic Computer Troubleshooting Skills (BCTS). The foundational proficiency level was selected for the courses given the need to build foundational abilities before advancing to specialized skills (58). The module content informed by the corresponding DigCom 2.2 competence was collaboratively created using resources from the Ministry of Health (MoH), implementing partners, the Ministry of Communications, Technology, and Innovations (MoCTI), and credible online sources. To address linguistic diversity among the health workers, language and multimedia enhancements in the widely spoken Krio language were incorporated into the modules (25).

**Table 2:** Mapping identified training needs to DigCom 2.2 digital competence framework.

Training needs based on TNA	DigCom 2.2 competence area	Proficiency level	Competence	Corresponding Training Module
Identify computer parts, desktop icons and how to use them.	Information and Data Literacy	Foundational	Managing data, information, and digital content	BCLIS Module 1 (A–E), 2
Learning simple methods to organize, store, and retrieve data and digital content using the home screen, desktop icons, and task bar				
Creating a digital identity, setting up and using email, composing and sending messages, and understanding common email terminology	Communication and Collaboration	Foundational	Managing digital identities	5, 6, 7
Creating and editing simple documents, including typing, saving, locating, and opening files	Digital Content Creation	Foundational	Developing digital content	3, 6
Securing devices and data, practicing safe online behaviors, identifying security threats, and knowing appropriate actions to protect digital content	Safety	Foundational	Protecting devices, personal data and privacy	4
Addressing basic computer hardware and software problems effectively	Problem solving	Foundational	Solving technical problems while operating device	BCTS Modules 1–10

### Training outcomes

Pre and Post-Test questionnaires were administered to the trainees for both BCLIS (*n* = 139) and BCTS (*n* = 142). Test scores for each trainee were recorded and categorized into the six level of understanding or competency (L1-L6) using the class interval method. Each level of understanding was then allocated corresponding points as follows: “No understanding – 1”, “ Very Low understanding – 2”, “Low Understanding = 3”, “Average understanding = 4”, “High level of understanding = 5”, “ Very high (Advanced) level of understanding = 6”. Therefore, the maximum point allocated for each level is 6 and the minimum point is 1. The percentage of scores for “No”, “Low”, “Average” and “High” levels of understanding for each course was calculated and compared between pre and post tests to determine competency gain (Table 3).

**Table 3:** Pre-and post-training competency levels of frontline health workers in digital literacy courses.

Competency Level	Score Interval	Description	No of trainees (Pre-Test)	Maximum Score (Pre-Test)	Score % (Pre-Test)	Pre-test Mean (SD)	No of trainees (Post-Test)	Maximum Score (Post-Test)	Score % (Post-Test)	Post-test Mean (SD)	Statistics
Basic Computer Literacy and Internet Skills Course											
L1	0–5	No understanding	0	0	0	3.1 (0.8)	0	0	0	4.9 (0.9)	*t* = −47.10, *p* < 0.001, *d* = 2.1, CI [1.8, 2.4].
L2	6–10	Very low understanding	26	52	12		1	2	0.3		
L3	11–15	Low understanding	78	234	53.9		11	33	4.8		
L4	16–20	Average understanding	27	108	24.9		24	96	14.1		
L5	21–25	High understanding	8	40	9.2		67	335	49.1		
L6	26–30	Very high understanding	0	0	0		36	216	31.7		
**Total**			**139**	**434**	**100**		**139**	**682**	**100**		
Basic computer troubleshooting skills course											
L1	0–5	No understanding	1	6	1.2	3.6 (0.9)	0	0	0	4.8 (0.8)	*t* = −24.32, *p* < 0.001, *d* = 1.3, CI [1.1, 1.6].
L2	6–10	Very low understanding	12	24	4.7		0	0	0		
L3	11–15	Low understanding	59	177	34.9		10	30	4.4		
L4	16–20	Average understanding	53	212	41.8		37	148	21.8		
L5	21–25	High understanding	14	70	13.8		69	345	50.8		
L6	26–30	Very high understanding	3	18	3.6		26	156	23		
**Total**			**142**	**507**	**100**		**142**	**679**	**100**		

**Figure 2:**
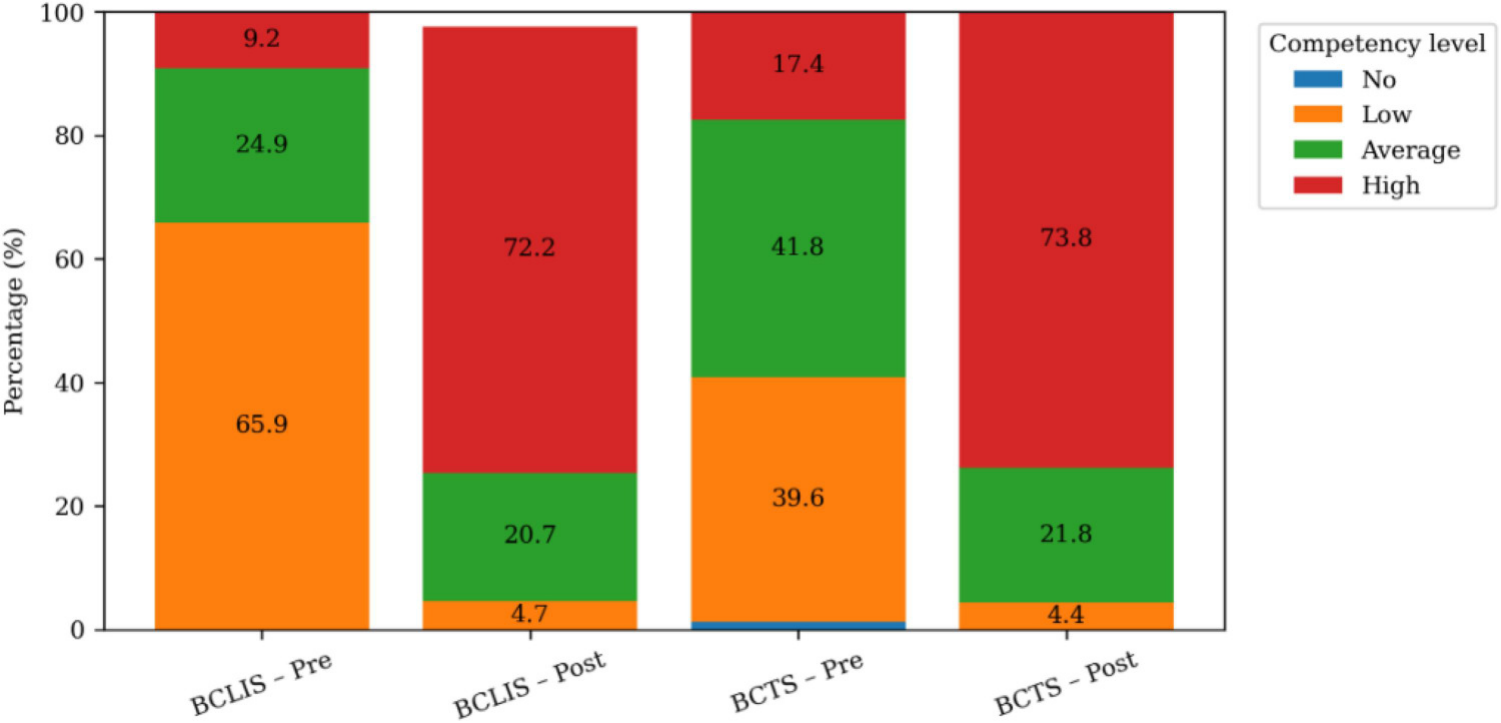
Improvement in digital literacy competency levels among health workers before and after BCLIS and BCTS training.

Our comparative analysis of pre and post-tests shows a significant shift in the level of understanding between across both training courses (Figure 2). In the BCLIS course, “High or Very high level of understanding” (levels L5–L6, scores 21–30) significantly rose from 9.3% to 80.8%, while “Low or Very low level of understanding” (levels L2–L3,scores 6–15) also significantly declined from 65.9% to 9.2% between pre and post-test scores. Similarly, in the BCTS course, “High or Very high level of understanding” increased from 17.6% to 73.8%, with “Low or Very low level of understanding” dropping from 39.6% to 4.4%. Mean scores (SD 0.9) improved from 3.1 to 4.9 and 3.6 to 4.8 for both BCLIS and BCTS courses respectively. Post-training satisfaction was high (95%).

Statistical analysis of the training scores demonstrated significant and practically meaningful gains. Paired *t*-tests confirmed strong evidence of improvement in both BCLIS (*t* = 47.10, *p* < 0.001) and BCTS (*t* = 24.32, *p* < 0.001). Large effect sizes for BCLIS (Cohen's *d* = 2.1) and BCTS (Cohen's *d* = 1.3) reinforce the practical impact, with narrow confidence intervals indicating robust findings. These results underscore the effectiveness of targeted, context-appropriate digital literacy interventions for enhancing health workforce capacity in resource-limited settings.

### Change management

**Figure 3:**
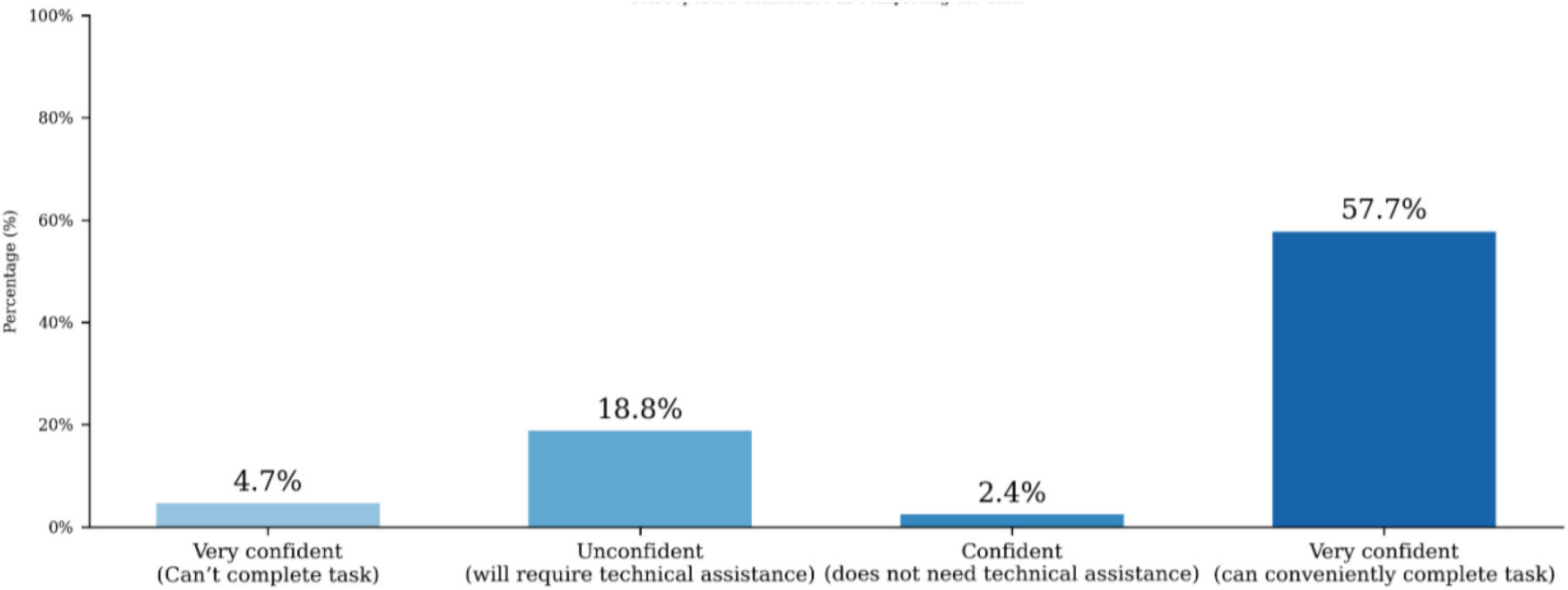
Change in self-reported confidence levels of health workers following digital literacy coaching.

Coaching was conducted onsite and was performance-based, with a focus on using the eSMT application to address key digital literacy challenges identified during the visits. Coaches reported satisfaction with the overall performance in 70% of the facilities visited. Following two rounds of coaching, self-assessment confidence levels among participants (*n* = 71) were 23.5% unconfident, 2.4% confident, and 57.7% very confident (Figure 3). This reflects a substantial reduction in the proportion of unconfident staff, dropping from 82.8% pre-training to 23.5% post-training.

Despite this improvement, the remaining 23.5% of participants who still felt unconfident likely reflect residual skill gaps and contextual barriers such as limited prior digital experience. Also, coaches reported inconsistent access to functioning devices in some health facilities. These findings suggest that continuous mentoring, peer learning, and periodic refresher sessions are essential to consolidate digital competence and ensure long-term behavioral change.

## Discussion (lessons learned)

### Digital health transformation and the need for early assessment of workforce readiness

A new digital culture is emerging in the health sector, compelling health workers to transition rapidly from manual, paper-based systems to digital platforms and data-driven workflows. This transition, however, is often constrained by limited e-Readiness, a key determinant of organizational and individual capacity to adopt new technologies. Previous studies identified inadequate readiness as a major factor behind failed digital health implementations (12). Our findings revealed that 82% of frontline health workers had low confidence levels in using digital technologies.

From the lens of the Technology Acceptance Model (TAM), such low self-efficacy negatively influences both perceived ease of use and perceived usefulness, two fundamental predictors of adoption behavior. The observed gaps therefore highlight the mediating role of digital readiness and self-efficacy in determining successful technology uptake. To address these factors, we employed a Training Needs Analysis (TNA) approach to align competencies with expected job tasks (21) and to build readiness prior to system deployment, consistent with capacity-building frameworks that emphasize pre-implementation user preparedness (43).

### Localizing relevant content for effective digital literacy capacity building

The design of digital literacy interventions must transcend generic computer training to embed local realities and learning contexts. Aligning training programs solely with institutional goals is insufficient for meaningful behavioral change. Capacity building theory suggests, contextual relevance and experiential learning drive sustained knowledge retention and confidence (25, 32, 38). In our study, locally adapted content, multimedia learning in the Krio language, and blended delivery methods significantly enhanced competence gains.

Drawing on TAM and self-efficacy theory, the incorporation of interactive and accessible learning modalities increased users' perceived ease of use and sense of mastery, both of which function as mediators between training exposure and sustained digital application. Low-cadre health workers, previously constrained by anxiety or unfamiliarity, reported improved confidence and autonomy in operating digital tools, reinforcing the importance of designing interventions that build both technical skill and psychological readiness (10).

### From digital competence to digital use: sustaining transformational change

True digital transformation extends beyond competency acquisition to consistent, confident digital use supported by enabling environments. Within the TAM framework, transformation occurs when perceived usefulness translates into actual behavioral intention and habitual usage. Our findings, where 60% of participants had their first computer experience during training, underscore the necessity of coupling skill acquisition with institutional support mechanisms such as device provision, connectivity, and peer mentorship.

Here, institutional support operates as a moderating factor that strengthens the link between digital literacy and technology adoption. Without such supportive conditions, even well-trained users may fail to translate competence into regular use. This reinforces the multidimensional nature of digital transformation, requiring synergy between individual capabilities and systemic enablers within the broader e-Readiness ecosystem (2).

### Digital literacy coaching a mechanism for change management

Beyond formal instruction, the project integrated continuous coaching and virtual mentorship, reflecting principles of change management theory that emphasize iterative support during technology adoption. Simulation-based learning and individualized coaching promoted adaptive learning and helped overcome resistance, a common barrier in digital transitions (45).

From a theoretical standpoint, ongoing coaching acts as a reinforcement mechanism that sustains self-efficacy and mitigates the decay of perceived behavioral control over time. This aligns with capacity-building models emphasizing cyclical learning, reflection, and reinforcement to consolidate digital transformation.

### Collaborative approaches for scaling and sustaining digital adoption

Collaboration with Ministry of Health partners facilitated the institutionalization of foundational digital literacy training as a prerequisite for deployment of specialized digital health applications. This systemic integration supports organizational e-Readiness by embedding human capacity development within national digital transformation strategies.

By situating these findings within the TAM and capacity-building frameworks, the study demonstrates that digital literacy training alone is insufficient. Sustainable adoption requires reinforcing mediating factors (self-efficacy, digital readiness) and moderating supports (institutional commitment, infrastructure). The intervention thus contributes new knowledge to the field by illustrating how a theoretically grounded, context-specific digital literacy program can accelerate workforce-level adoption and system-wide digital transformation in LMICs.

### Theoretical and practical implications for digital health adoption

This study shows that a competency-based, contextually adapted, and blended digital literacy intervention effectively improved technical and behavioral adoption of technology. Unlike traditional lectures, this approach increased participants' self-efficacy and perceived relevance, core aspects of the Technology Acceptance Model (TAM). Digital literacy emerged as a key mediator between workforce readiness and technology adoption, shaping perceived usefulness, ease of use, and intent to adopt. The research also highlights the moderating roles of e-readiness, institutional support, and self-efficacy. Although promising, long-term impact remain unclear since the project is still ongoing as well as limitations in the study design. Nonetheless, the approach offers a scalable model for LMICs, underscoring the need for integrating ongoing learning, mentorship, and monitoring into national digital transformation strategies.

However, caution should be exercised in generalizing these findings to settings outside Sierra Leone. Cultural differences, such as language preferences, attitudes towards technology, and workplace norms, may influence the effectiveness of digital literacy interventions in other countries. Moreso, the geographic coverage is limited to only the 4 Districts involved in the DIPC project. Additionally, variations in technological infrastructure—such as internet connectivity, device availability, and IT support—and differences in the baseline educational levels of health workers could affect both the implementation and outcomes of similar programs elsewhere. Addressing these contextual factors may require adapting training content, delivery methods, and support mechanisms to local needs. Future research should focus on evaluating the transferability of this model across diverse environments, systematically exploring how cultural, infrastructural, and educational variables mediate or moderate the impact of digital literacy capacity-building interventions.

## Limitations and constraints

This paper is formative and does not cover long-term training outcomes and impacts of the adopted approaches since coaching and mentorship activities are still ongoing. It highlights potential bottlenecks and proposes mitigation strategies to enhance the adoption of digital skills and prevent unsuccessful digital health implementations in a typical low- and middle-income country (LMIC). Moreover, the lack of a comparison group, reliance on self-reported competence, and absence of longitudinal follow-up limit the ability to draw strong causal inferences or predict sustained impact.

Recognizing these limitations, a follow-up evaluation has been planned to assess the retention of digital competencies and sustained application of learned skills over time. This will determine knowledge retention, continued system use, and factors influencing long-term digital adoption. Further summative evaluation will therefore be required to measure knowledge gain trajectories, learning curves, and the overall outcome of eSMT application in vaccine logistics management.

## Conclusion

This formative study underscores the critical importance of contextually adapted, competency-based digital literacy interventions for advancing digital health adoption in LMICs. While initial findings indicate positive impacts on workforce readiness and technology uptake, the absence of long-term outcome data and a comparison group limits the strength of causal inferences. Ongoing coaching and mentorship, as well as planned follow-up evaluations, are essential for assessing knowledge retention and sustained digital competency. The lessons learned highlight the need for flexible, scalable strategies that consider local cultural, infrastructural, and educational factors, in line with global best practices. Future research should focus on rigorous, longitudinal evaluations to establish generalizability and inform evidence-based policy and practice in diverse settings.

## Data Availability

The original contributions presented in the study are included in the article/Supplementary Material, further inquiries can be directed to the corresponding author.
