# Supplementary figures and images for: Foundational digital literacy training for frontline immunization officers: lessons from implementing the electronic stock management tool across selected comprehensive health centers in Sierra Leone
Source: Front Digit Health. 2025 Dec 19;7:1673085. doi: 10.3389/fdgth.2025.1673085 (PMC12757871; doi:10.3389/fdgth.2025.1673085)

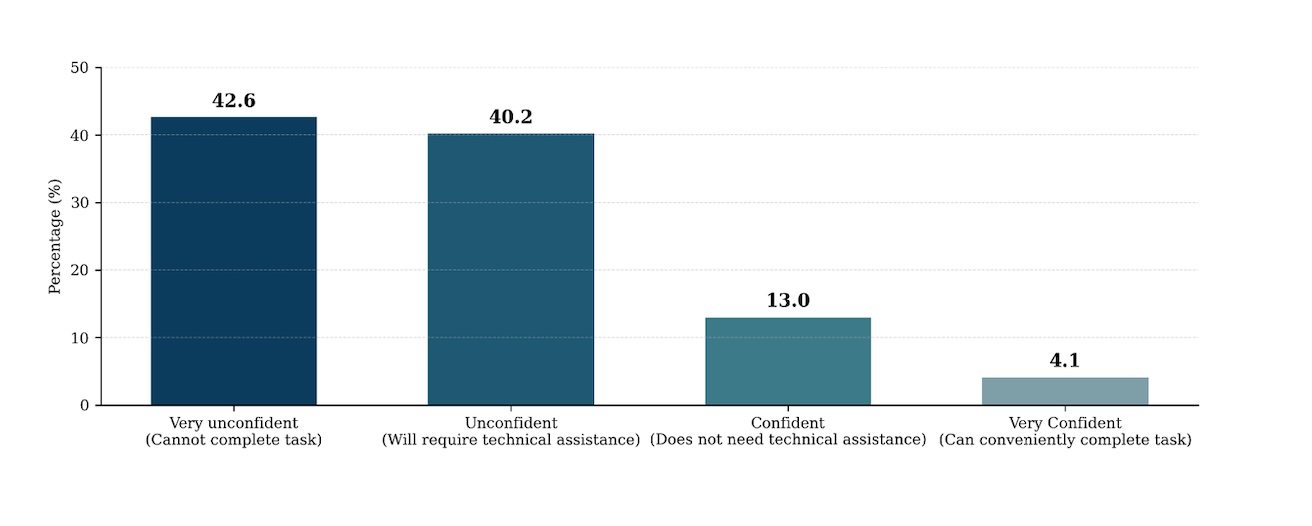

Supplement: Supplementary file 1 [file Image1.tiff]

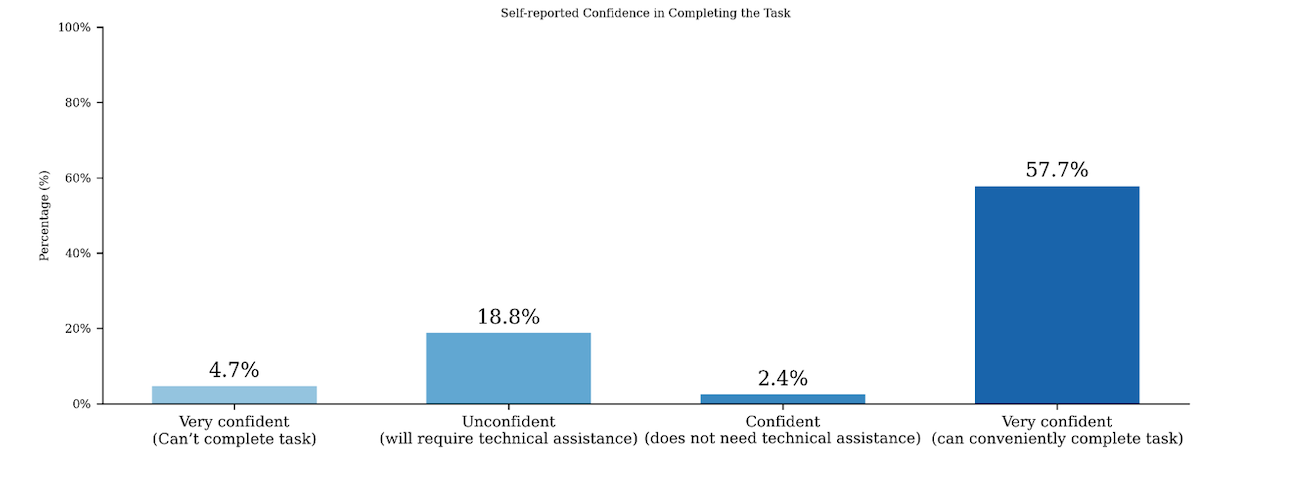

Supplement: Supplementary file 2 [file Image2.tiff]
